# Malignant Clinical Presentation of a Benign Granular Cell Tumor of Breast in a Patient with Previously Treated Contralateral Invasive Ductal Carcinoma

**DOI:** 10.1155/2012/974740

**Published:** 2012-11-29

**Authors:** Deniz Tural, Emre Akar, Tülin Öztürk, Hande Turna, Süheyla Serdengeçti

**Affiliations:** ^1^Division of Medical Oncology, Department of Internal Medicine, Cerrahpasa Medical Faculty, Istanbul University, 34098 Istanbul, Turkey; ^2^Cerrahpasa Medical Faculty, Istanbul University, 34098 Istanbul, Turkey; ^3^Department of Pathology, Cerrahpasa Medical Faculty, Istanbul University, 34098 Istanbul, Turkey

## Abstract

GCT is a rare neoplasm and usually shows the benign character. GCT can occur in any body site and may be multifocal. The most common involved site is tongue which accounts for nearly 30% of all cases but skin and subcutaneous tissue are also affected frequently. Breast is an unusually involved site and accounts for 6% of all GCTs. The histiogenesis of GCT is still controversial but further investigations and immunohistochemical examinations were exposed to neural origin and the tumor is thought to be derived from Schwann cells of peripheral nerves. Generally used technique to diagnose GCT is the positivity of S-100 immunohistochemical staining. Despite its benign nature, GCT may mimic breast carsinoma clinically and radiologically and easily be misdiagnosed for breast cancer. We herein report a case of granular cell tumor that arose in a 56 year-old female patient who previously had been treated from an invasive ductal carcinoma in contralateral breast.

## 1. Introduction

Granular cell tumor (GCT) was first described by Abrikossoff in 1926 [1]. GCT is a rare neoplasm and usually shows the benign character. Malignancy is extremely rare and comprises less than 2% of GCTs [2]. The tongue is the most common site of GCT that is occasionally located in breast. Approximately 6% of GCTs occur in the breast [3]. Despite its benign nature, GCT may mimic breast carsinoma clinically and radiologically and easily be misdiagnosed for breast cancer. Misdiagnosis of malignancy may cause to unnecessary radical treatments. Thus, there is a challenge to distinguish GCT from breast cancer based on clinical and radiological findings. This challange may be more difficult when GCT seems in a patient who has previously or simultaneously breast cancer such as invasive ductal carsinoma (IDC). 

We herein report a case of granular cell tumor that arose in a patient who previously had been treated from an invasive ductal carcinoma in contralateral breast.

## 2. Case Report

**Figure 1:**
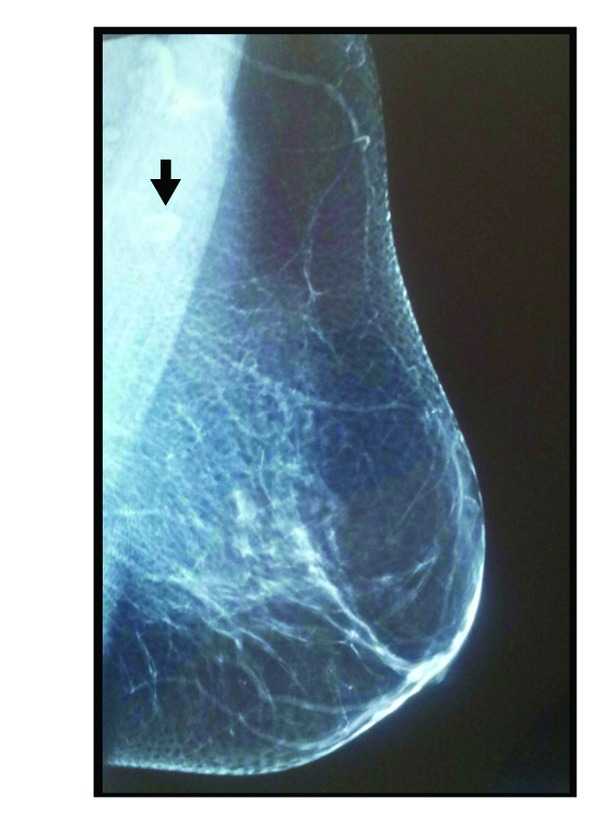
A mammography image with 18 × 10 mm measured mass lesion that localized in the left axillary region; superposed to pectoral muscle and have irregular, indistinct margin.

**Figure 2:**
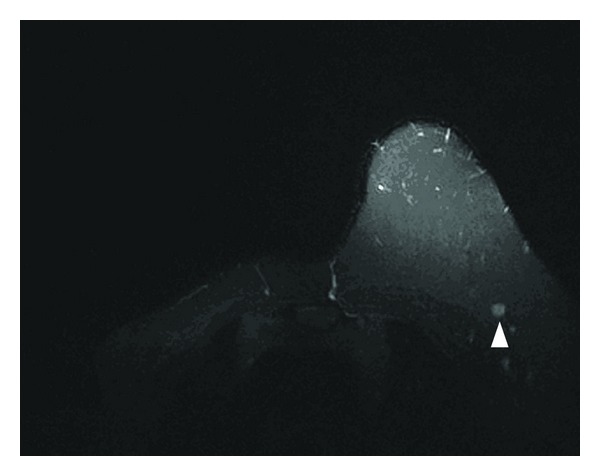
An MRI image demonstrates an axillary reticular mass lesion with T1A hypointense signal intensity changes.

**Figure 3:**
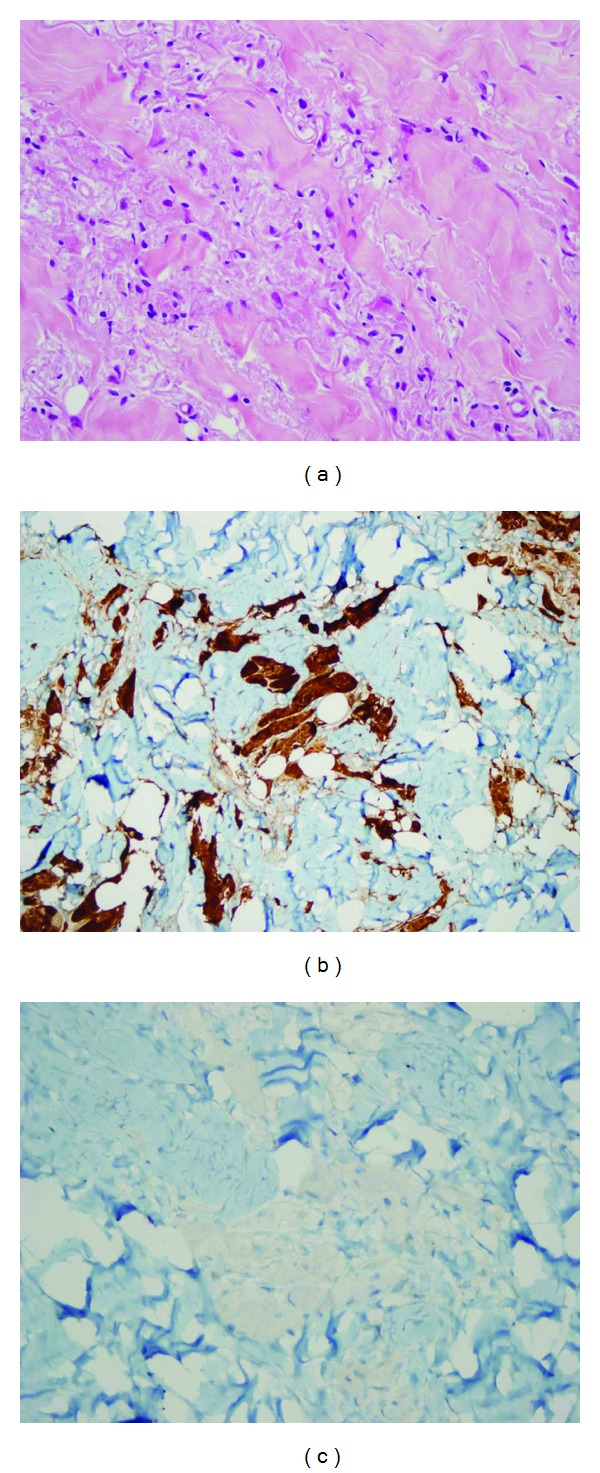
Pathological microscopic examination demonstrates the infiltration of cells with eosinophilic granular cytoplasm (a); (hematoxylin eosin, ×400), immunohistochemical staining positive for S100 protein (b); (×200), and negative for cytokeratin (c); (×400).

A 56-year-old female presented with a mass in her left breast that detected on a routine mammogram. She had been invasive ductal carsinoma in her right breast 3 years ago and modified radical mastectomy has been performed subsequently. In a routine follow-up mammography breast ultrasound imaging showed a mass in the upper outer quadrant and near to axillary tail of her left breast which measured 18 × 10 mm (Figure 1). Mass appeared to be attached to the underlying pectoral muscle. Magnetic resonance imaging (MRI) was performed to the left breast and a solid mass was confirmed (Figure 2). Given her IDC history, the mass was very suspicious of malignancy. The morphological and immunohistochemical examinations of core biopsies revealed a granular cell tumor with S-100 positivity and cytokeratin negativity (Figure 3). Lumpectomy was performed with wide local healthy tissue excision and there was no tumor cells within 10 mm of the margin. The postoperative period was uneventful and the patient is well and free of disease after 3 months of operation.

## 3. Discussion

When Abrikossoff initially described granular cell tumor, it was decelerated that the tumor had myogenic origin and name of granular cell myoblastoma due to their cytological similarities to myogenic cells [1]. The histiogenesis of GCT is still controversial but further investigations and immunohistochemical examinations were exposed to neural origin and the tumor is thought to be derived from Schwann cells of peripheral nerves [4]. Generally used technique to diagnose GCT is the positivity of S-100 immunohistochemical staining this is one of the lines of evidence to support Schwann cell origin of GCT. In addition, GCT cells share many similarities with Schwann cells on electron microscopy [5].

GCT can occur in any body site and may be multifocal. The most common involved site is tongue which accounts for nearly 30% of all cases but skin and subcutaneous tissue are also affected frequently. Breast is an unusuall involved site and accounts for 6% of all GCTs [3]. GCT prevalence is approximately 1 : 1000 cases of breast cancer. GCT of breast is largely a disease of females in the 4th to 6th decades who are commonly premenopausal but 6.6% of all cases consist of male patients [6]. 

Breast carcinomas are frequently localized in the upper outer quadrant. In contrast, GCTs of breast commonly occur in the upper inner quadrant of the breast and that appears to parallel the distribution area of the supraclavicular nerve [7]. 

Clinical presentations of breast GCT are 70% of cases detected by palpation, 26% through screening, and 4% during followup after breast malignancy. The mass features are various and described as firm and painless or as elastic consistency with breast pain. Generally the masses are mobile but may be fixed to the pectoralis muscle [6].

Colocalization and coexistence with IDC has been reported in previous publishings [3, 8]. In these cases, GCT is localized in the same breast with IDC and found as pathological incidences in mastectomy specimens for primary and recurrent IDC. There is one publishing that describes a patient who has IDC in one breast and GCT in the other, simultaneously [9]. In our case, GCT is determined in the left breast and she had IDC in the right breast previously. Contralateral existence of GCT and especially prior breast carcinomas raises the question of whether there is a recurrence or primary malignancy because of the clinical and radiological similarities between GCTs and breast carcinomas. This problem emphasizes the clinical importance of distinguishing the two lesions. With the aim of preventing inappropriate and unnecessary radical treatments, GCT should be considered more in the diagnostic processes of the breast masses.
